# Mixed partisan households and electoral participation in the United States

**DOI:** 10.1371/journal.pone.0203997

**Published:** 2018-10-10

**Authors:** Eitan Hersh, Yair Ghitza

**Affiliations:** 1 Department of Political Science, Tufts University, Medford, MA, United States of America; 2 Catalist, LLC, Washington DC, United States of America; Augusta University, UNITED STATES

## Abstract

Research suggests that partisans are increasingly avoiding members of the other party—in their choice of neighborhood, social network, even their spouse. Leveraging a national database of voter registration records, we analyze 18 million households in the U.S. We find that three in ten married couples have mismatched party affiliations. We observe the relationship between inter-party marriage and gender, age, and geography. We discuss how the findings bear on key questions of political behavior in the US. Then, we test whether mixed-partisan couples participate less actively in politics. We find that voter turnout is correlated with the party of one’s spouse. A partisan who is married to a co-partisan is more likely to vote. This phenomenon is especially pronounced for partisans in closed primaries, elections in which non-partisan registered spouses are ineligible to participate.

## Introduction

Building upon recent research on assortative mating, partisan preferences in romantic partners, and, more generally, affective polarization [1–6], this article analyzes a national database of households of registered voters to describe a.) the rate at which Democrats and Republicans in the United States are intermarried, b.) the demographic and geographic correlates of partisan intermarriage, and c.) the relationship between partisan intermarriage and electoral participation.

Our study furthers our understanding about the nature and consequences of partisanship, social networks, and mass political behavior. By leveraging millions of voter registration records, we are able to make discoveries that would not be possible when using traditional survey techniques. Rather than investigating a random sample or convenience sample of individuals, we study a population of households, for which we have records of all registrants within the household. By studying population data, we are able to paint a highly detailed portrait of the population of interest, such as by studying partisan intermarriage among particular age groups or in particular neighborhoods. By studying government records, we are able to measure electoral participation without concern about survey misreporting.

Our empirical investigation has two components. First, we observe the rate of partisan intermarriage and how that rate varies on three dimensions: gender, age, and location. Then, we study the relationship between partisan intermarriage and electoral participation. We ask whether voters in mixed partisan households are less likely to vote or more likely to vote than similar voters in single-party households. And we interpret how the observed patterns shed light on competing theories of behavior.

### Location, gender, and age

Gender, age, and geography each bear strong and well-studied relationships to partisan identity. Observing how these relationships operate within marriages is important because average effects often can mask heterogeneous effects that have critical implications for understanding political behavior. For instance, many scholars have studied, in detail, how partisan voters are situated geographically (e.g. [7–9]). Suppose we see a number of districts in which half the votes go to Democrats and half go to Republicans. Our interpretation of those districts would be quite different if the houses were split evenly between Democrats and Republicans versus if the voters *within those houses* were split evenly. But prior studies of partisan geography, which focus either on aggregate units like precincts or else on individuals, pass over this essential social unit in which citizens are situated.

In studying geography, what do we expect to find? As Chen and Rodden emphasize [10], Democrats tend to be more clustered together than Republicans. Accordingly, we suspect that mixed-partisan households are least prevalent in highly Democratic urban cores and are most prevalent in areas of mixed partisanship. But outside of the few homogenous partisan areas, we have no a priori expectation about the strength of the relationship. It could be that the level of partisan marriage tracks closely with the partisanship of a geography or hardly at all with it.

### Gender

There is a well-known gender divide in American politics: men are more likely to be Republican than women. In 2016, 42% of women versus 53% of men voted for President Trump. A sharp gender gap is also present when partisan identification, rather than party voting, is the target of study [11]. We suspect that the gender gap exists even conditional on household partisanship. In mixed partisan heterosexual married households, we assume the male partner is more likely to be Republican and the female partner is more likely to be Democratic. We expect this to be true even in households in which one partner is an independent. That is, we expect that Pr(*I_M_*|*D_F_*) > Pr(*I_F_*|*D_M_*), where *I* represents independents, *D* represents Democrats, and subscripts denote gender.

Of course, a gender gap could theoretically exist in the electorate but not exist conditional on household partisanship. This could occur if very few partisans intermarry or if marriage rates are conditional on gender and partisanship.

### Age

A feature of political party identification in the United States is that younger voters are more likely to identify as independents than older voters. In the 1968 National Election Study survey, 42% of respondents under age 35 identified as independent compared to only 17% of respondents 65 years and older (a 25 percentage point difference). Forty years later (when the cohort of younger voters became the cohort of older voters), 51% of NES respondents under 35 were independent compared to 30% of respondents over 65 (a 21 percentage point difference). While a full decomposition of the age, period, and cohort effects is outside the scope of this analysis, the cross-sectional time series data does suggest that multiple mechanisms may be at play. Looking at a single, current snapshot of married households, what do we expect to find? We expect that among younger households, there will be many more unaffiliated registrants, and this is likely to result in a smaller share of Democratic-only and Republican-only households than among older voters. Again, the relationship between age and partisan independence need not hold within household, but as with gender, our base expectation is that it will.

### Voter turnout

Controlling for individual age, race, gender, party, and state, will turnout among mixed-party couples be higher or lower than same-party couples? One reasonable hypothesis is that a person who is less interested in politics (and less likely to vote) might just go along with the party identification of their spouse [12]. Partisan mixed-marriage could be correlated with higher levels of political engagement because it signals a desire to identify in a particular way even if it means a conflict with one’s spouse. In other words, in mixed-partisan marriages, politics is important enough that individuals do not go along to get along. If it’s that important, perhaps the individuals are more engaged in politics than the average citizen.

However, there are at least two rationales for thinking that mixed-partisanship might have a depressing effect on turnout. First, participation is correlated with partisan intensity [13] and intense partisans are probably less likely to be in mixed- partisan households to begin with [1]. Second, partisans may react to being at odds with their spouse by withdrawing from political participation [14]. Voters may want to avoid political debates that surround election participation or they may employ a logic that they would cancel out their spouses vote and that this makes voting a waste of time.

### A note on descriptive research

Descriptive research and causal research are both important parts of the scientific process. Determining whether, how, and in what circumstances a phenomenon occurs can help explain why the phenomenon occurs. Similarly, if one has a theory about why a phenomenon occurs, it can help explain whether and how it occurs. Descriptive hypotheses and causal hypotheses go hand-in-hand. And yet, quantitative political science research has grown almost unaccustomed to descriptive research.

There is a reason for this. In the first generation of the mass survey, scholars made important scientific progress by describing basic rates and kinds of behaviors of the public. Appropriately, this research was followed by scholarship looking beyond the descriptive questions and utilizing randomization and natural experiments to answer ‘why’ questions. The causal research benefited from—indeed, depended on—a strong foundation of descriptive evidence.

Today, new data sources permit us to discover a rich new set of empirical phenomena. These datasets allow us to perceive the state of the world in a much higher resolution than a small survey allows. Naturally, the first step in the scientific process is to discover and carefully document key phenomena of interest. Once we have a firm handle on these discoveries, it is then possible to dig in deeper to causal questions.

In the present study, we aim to describe a phenomenon—the attributes and behaviors of mixed partisan households—that would be very hard to see in other sources of data. We ask questions like: How many mixed-partisan couples are there? What are their demographic characteristics? Where, geographically, are they concentrated? Do they vote less than same- party couples? Answering these questions allows us to weigh in on important theoretical questions pertaining to the nature of partisanship, social networks, and voting.

But to readers who are narrowly interested in causal hypotheses, it may be frustrating that we cannot, for example, follow up our discovery of diminished turnout rates in mixed-partisan marriages with a test of causal processes. For two Republicans of the same age, gender, race, and state, why is the one married to a Democrat ten percentage points less likely to vote than the one married to a fellow Republican? We do not know. We suspect, of course, that it is some combination of less politically active people marrying outside their party as well as a direct effect of cohabitation. But since researchers cannot randomly assign marital partners, causal questions in this line of inquiry are difficult to answer.

Even without a clean causal explanation for the evidence we describe, the evidence itself is nevertheless useful. It lays an important foundation for future work. For example, there are a number of well-studied phenomena (e.g. turnout, political geography, the partisan gender gap) that may have previously-unknown heterogeneity lurking beneath them. For instance, we discover here that mixed-partisan neighborhoods have high rates of mixed-partisan households. This is not an obvious finding and may be useful to those designing studies on the effects of neighborhood on attitudes or behaviors. In general, our findings are important for future researchers because the most common and essential social network that can influence citizens–the family unit–is usually absent in the study of individuals. Our purpose here is to get the facts straight about this family unit. Doing so—and doing so carefully—is a fundamentally important part of the scientific process.

## Materials and methods

Our data come from Catalist, a data firm that manages a national database of voting- aged people, with a particular focus on registered voters and political/civic data. Catalist’s data includes regularly-updated voter registration records from every state. On account of Catalist’s comprehensive voter data, numerous scholars have recently employed Catalist for studies of political participation in the U.S. (e.g. [15–17]). For this study, the complete dataset of voter registration records provides a key asset. All registered voters are identified with a residential address. By observing multiple registrants at the same address, we can study household attributes and behaviors in ways that would be difficult with standard survey techniques, as surveys are not typically administered to complete households.

In order to leverage the detailed information about households that is contained in voter registration data, we must define a set of married couples. In our main analysis, we focus on pairs of individuals who:

- are composed of one male and one female,
- are registered to vote at the same address,
- share a surname,
- are within fifteen years of age from one another (to avoid counting mother-son and father-daughter pairs as couples),
- are the oldest such pair registered in the household (to avoid counting siblings who are registered in their parents’ home as married).

The analysis excludes households with more than ten registered voters. These households are likely to be apartment buildings or dormitories misclassified in the voter registration records as single household units.

The overwhelming majority of heterosexual married couples in the United States share a common surname [18]. According to a 2015 analysis by the New York Times, over 80% of married women have taken their husbands’ names. Obviously, married women who keep their maiden names are not a random subset of married women. Older women are slightly more likely to take their husband’s name (86% of women first married before 1970 changed their name compared to 78% of women married in the 2010s). Wealthier and more urban women are less likely to change their name. And there are religious/cultural differences. According to the New York Times analysis of its published wedding announcements, for instance, two-thirds of Catholic women took their husbands names compared to one-third of Jewish women [19].

To assess bias in our analysis presented from our definition of marriage, we run our entire analysis through thirty-two variations of the marriage definition. These variants relax our set of restrictions. We allow for couples to have different surnames. We allow for same-sex pairs. We allow for pairs who are more than fifteen years apart. Rather than restricting to the oldest couple in a household, we choose random pairs from households. We do not restrict to households with fewer than ten people. These variants are allowed one at a time and in concert, totaling 25 variants.

As we relax restrictions from those in our main analysis, we are more likely to include a diverse array of actually-married couples (e.g. same sex marriages, married couples in which both partners retained their surnames). However, we are also more likely to count many non-married people as married (e.g. two twenty-something male roommates with different last names would be counted as married if we relax the opposite-sex and same- surname restrictions). The effect of this is that with fewer restrictions, the married couples as a whole appear less Republican. Republicans are likely to form a greater share of the “traditional” opposite-sex/same-surname couples than they are of the couples who also include same-sex and different-surnamed couples. We will show how variations in definitions affect our results. While the overall composition of married couples changes with these restrictions (in the Democratic direction), the overall patterns *within* the subset of married couples are not particularly sensitive to the different definitions.

An unrelated source of bias inherent in our analysis stems from the fact that we focus only on pairs if both partners are registered to vote (and thus have a recorded party affiliation). It is possible that one’s decision to register to vote may be affected by whether he or she is married to a co-partisan. If true, this might cause us to underestimate the number of mixed partisan households. This source of bias has an obvious analog in the survey setting. As is well-known, due to misreporting and selection bias, survey samples can dramatically over-represent the political engagement of the public. In both the case of government records and surveys, such threats to inference imply that interpreting the data requires appropriate caution.

For the married couples in our analysis, we utilize a series of public records to test our hypotheses. First, we utilize party registration records. In 31 U.S. states, most registered voters are listed with a political party affiliation (typically Democratic or Republican) or else are independent. Here, we count independents and minor party registrants together, under the label of ‘other.’ The states that collect party registration records are quite representative of all U.S. states [9]. Furthermore, as Hersh and Nall note, party registration is very highly correlated with party identification. In the 2008 CCES, which was matched to voter records, 95% of registered Democrats self-identified as Democratic and 96% of registered Republicans self-identified as Republican.

Note that party registration states do vary in whether they allow independents to vote in primaries. Whether a state opens or closes its primaries to independents correlates with the rate at which individuals register with a party. We deal with this problem in several ways. First, we have tested whether our results, particularly our turnout results, differ in open and closed primary states. We show the comparison below. Second, in many states, the state publicly records whether individuals voted in Democratic or Republican primaries. Thus, in many states that allow independents to vote in primaries, we can measure in which party’s primary they voted. This permits us to alternatively define partisanship based on a combination of party affiliation and the primary voting patterns of independents. This alternative definition does not affect our results. Third, on account of sufficient data, we can examine patterns within states and employ fixed effects. Our results are robust within states.

For each individual, we utilize public records of their gender and age. In a small number of cases where age or gender are missing in the public record, Catalist substitutes commercial predictions of these attributes. Individual race also comes from a combination of public records and inferred race where public records are unavailable. Catalist situates each voter in their Census block group and precinct. Precinct returns enable us to assess the partisan balance of the neighborhoods in which couples live. Finally, we utilize public records of electoral participation in primary and general elections, which are also available from voter registration records.

## Results

Of 105,935,400 registered voters in party registration states, 36,550,002 (35%) meet our main criteria for married couples. Our main analysis focuses on these 18,275,001 male-female couples.

Table 1 shows an initial view of these registered voters. Several observations can be made. First, the largest internal cells in this table are on the diagonal. Of all married households, 25% contain two Democratic partners, 31% contain two Republican partners, and 15% contain two independent partners. Altogether, 71% of registrants share an affiliation and 29% do not. Of the mixed households, a third are Democratic-Republican, a third are Democratic-independent, and a third are Republican-independent.

**Table 1:** Party composition of married households.

	Female			
Male	Democratic	Independent	Republican	Total
Democratic	25%	4	3	32
Independent	6	15	5	26
Republican	6	5	31	42
Total	37	24	39	100

Note: Observations: 18,275,001 married couples in party registration states.

In S1 Table, we show the rate of different pairings according to the thirty-two alternate definitions of marriage. For instance, if we include different-surname and different-sex couples in our analysis (row 6 of the table), 65% of the couples share an affiliation, compared to 71% in the main analysis. Ten percent are D-R (rather than 9% here). In fact, across all thirty-two variants of the marriage definition, the percent in D-R relationships never fluctuates by more than a percentage point or two.

In SI Text, we offer two approaches to evaluating how the observed rate of mixed partisan households relates to two different baseline expectations: a comparison to racially mixed households and a comparison to the partisan balance within age and geographic cohorts.

### Gender, age, and geography

#### Gender

Table 1 sheds light on how the partisan gender gap operates within households. Just as there is a partisan gender gap among individuals, there is a partisan gender gap among married couples. Looking at the marginal percentages, there is a five-percentage point difference in men and women registered as Democratic (37% for women, 32% for men). There are also twice as many Democratic-Republican households in which the husband is the Republican than in which the wife is the Republican.

Table 2 shows this imbalance more clearly as it offers conditional percentages. Among Democratic women, 67% of husbands are Democratic, but among Democratic men, 77% of wives are Democratic. Among Republican men, 73% of wives are Republican, but among Republican women, 79% of husbands are Republican. In other words, couples are more likely to share a party affiliation in households where the wife is a Republican or where the husband is a Democrat. This descriptive finding is especially important in light of the changing nature of household composition in the US. Over several decades, the number of single households has grown, especially for women [20]. A partisan gender gap might emerge in the population without it also emerging within married couples. However, our data show a partisan gender gap within couples.

**Table 2:** Party affiliation, conditional on spouse’s party affiliation.

	Female			
Male	Democratic	Independent	Republican	Total
Democratic	77%	12	11	100
	67	16	9	
Independent	24	57	19	100
	17	61	13	
Republican	14	13	73	100
	16	23	79	
Total	100	100	100	

Note: Observations: 18,274,446 married couples in party registration states. First row represents row percentages (female conditional on male). Second row represents column percentages (male conditional on female).

#### Age

**Fig 1:**
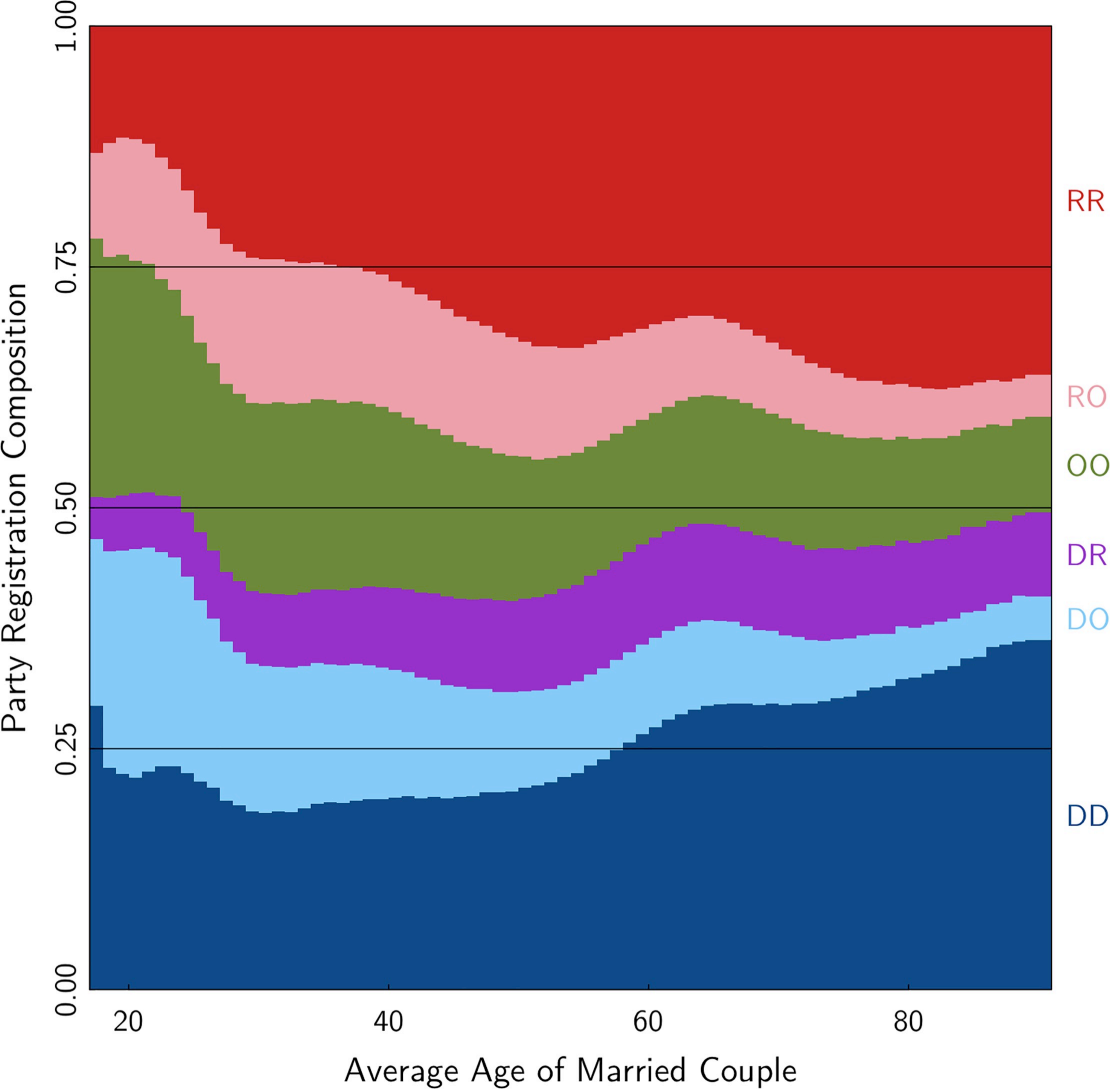
Household party composition, by age.

Next, we turn to the relationship between household partisanship and age. In evaluating age, it is worth noting that married couples, in general, are considerably older than the pool of all registered to voters (see S2 Fig. for a density plot comparing married couples to all voters). Fig 1 shows the six household types by age (‘O’ designates independents and other non-major-party registrants). There is a dramatic difference in the distribution of household types between the youngest cohort and the oldest one. Among married couples under 30, fewer than half (40%) are Democratic-Democratic or Republican-Republican. Among couples over 80, almost 71% are in D-D or R-R pairs.

Young voters not only have the lowest incidence of D-D and R-R households, they also have the lowest incidence of D-R households. As is visible, the purple area that represents Democrats married to Republicans almost doubles in width from twenty-somethings to octogenarians. Indeed, the R-R, D-D, and D-R lines are larger for older voters while the R-O, D-O, and O-O lines diminish.

To be sure, multiple mechanisms can lead to the pattern visible in Fig 1. One is simply a cohort difference between younger and older generations. It could be that the current younger generation is now and will always be both more independent and Democratic leaning than the current older generations. Ghitza and Gelman show persistence in partisan leanings based on how the parties were performing during one’s younger years [21]. For example, the pro-Democratic bump visible in Fig 1 for registrants around age 65 may be driven by political events from their teens and twenties, such as the Kennedy presidency and the Watergate scandal.

A second plausible mechanism is that, in every generation, younger voters identify as independent at higher rates than older voters. Young voters might still be figuring out their partisan identity. Or, younger independents may be closet partisans who just prefer to think of themselves as independents. Either way, in an aging story, the young couples containing political independents in Fig 1 will eventually identify as partisans, just like the current generation of older voters.

A third mechanism is a direct effect of cohabitation. A truly independent voter married to a partisan may be influenced by the partisan to join their team. While this surely can happen, it is worth noting that the green line in Fig 1, which represents couples who are both independent, diminishes over the age span in a similar fashion as the D-O and R-O couples. One might think if spouses are influencing each other, they might also influence each other to become independent. But the trend is away from independent registration.

While our data do not allow us to evaluate these competing mechanisms, the descriptive evidence presented is nevertheless important for laying foundations for future work. For example, similar to the note above regarding gender, a difference between young and old cohorts in the population might have its roots in differential rates of marriage between young and old cohorts, particularly in a time when young adults are delaying marriage compared to older generations. The evidence here suggests even within married couples, young and old cohorts have different compositions with respect to partisanship.

#### Geography

**Fig 2:**
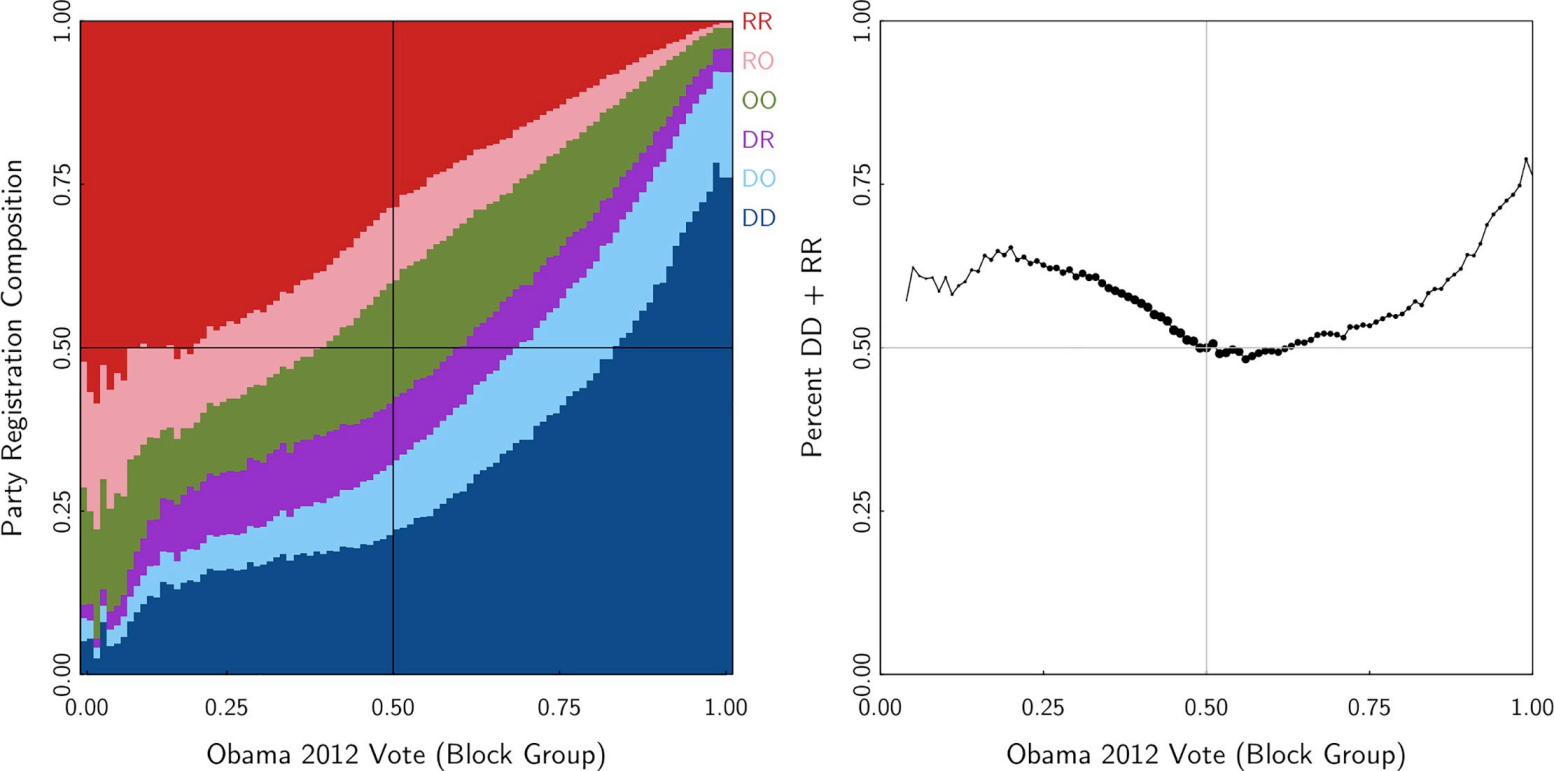
Household party composition, by neighborhood.

Our final exploration before examining voter turnout focuses on the relation- ship between household partisanship and neighborhood partisan composition. We situate each married couple in their Census block group, which we will think of as a neighborhood. Relying on Catalist’s link between block group and voting precinct, we are able to provide the block-group-level Presidential vote share for 2012. The x-axes in Fig 2 show the Obama percentage of the two-party vote.

There are two important features of Fig 2. First, the neighborhoods that are most divided between Democratic and Republican supporters have the highest number of mixed-partisan households, but the relationship is fairly modest. In a neighborhood where 66% of votes went to Obama, 9% of households are D-R. In a neighborhood where 33% of votes went to Obama, 10% of households are D-R. The right-side plot in Fig 2 illustrates the u-shaped relationship between the percent of households that are D-D and R-R and the percent of voters in the neighborhood supporting the Democratic candidate for president.

The second important feature of Fig 2 is the difference between the overwhelmingly Republican neighborhoods and the overwhelmingly Democratic ones. In neighborhoods that are overwhelmingly Democratic (e.g. 90%+ Democratic), 68% of households are D-D households. But in neighborhoods that are overwhelmingly Republican (90%+ Republican), only 55% are R-R. This phenomenon reinforces the point made by Chen and Rodden about partisan geography [10]. Most of the overwhelmingly Democratic neighborhoods are homogenous African-American neighborhoods. Among African-Americans, support for Democrats in general and President Obama in particular is close to unanimous. The neighborhoods that vote overwhelmingly Republican, however, are more ideologically diverse. Even in these neighborhoods, there are significant number of registrants who are independents or who are married to independents.

For researchers interested in geographic political sorting [22], the focus is typically on individuals situated in neighborhoods. The descriptive finding here helps inform future work with the insight about the location of mixed-party households. Accounting for these households might help researchers design studies that accommodate this important source of heterogeneity within different kind of neighborhoods (e.g. by blocking on household type in experiments).

#### Voter turnout

Having established the basic relationship between gender, age, geography and mixed- partisanship, we now turn to our more detailed analysis of voter turnout. We examine voter turnout in the 2012 and 2014 general elections as well as in the primary elections in those years. We ask whether being married to someone of another party is associated with an increase or decrease in one’s participation in elections. Different theories predict different outcomes for this analysis, as discussed above. Again, the reason for the attention to turnout in spite of the fact that the analysis here does not offer a causal explanation for the observed findings is to discover key features of political behavior that can inform future studies. Many political scientists who study voter participation attend to variation in turnout by individual traits (e.g. race, gender, age) and in geographic traits (e.g. competitive locations), but often neglect household-level traits. By showing how household partisan composition correlates with turnout, we hope the analysis can inform future experimental and observational studies of turnout where heterogeneity across household types might affect conclusions.

In Table 3, we show individual voter turnout among registrants in the sample of married couples. In all elections we analyze, turnout is highest among Republicans and lowest among independents/others (represented by ‘O’ in the table). Democrats are in the middle. In the 2012 Presidential election year, when the Democratic president won reelection, the turnout gap between Democrats and Republicans was only three percentage points. In 2014, when Republicans did better, the turnout gap is seven percentage points.

**Table 3:** Turnout by individual party registration.

	Year: 2012			Year: 2014		
Party	Primary	General	Observations	Primary	General	Observations
D	28%	86	11,585,923	27	63	12,225,381
O	14	80	7,876,904	11	53	8,672,319
R	41	89	13,450,984	31	70	14,275,903

Note: Observations include all individuals in married couples.

**Fig 3:**
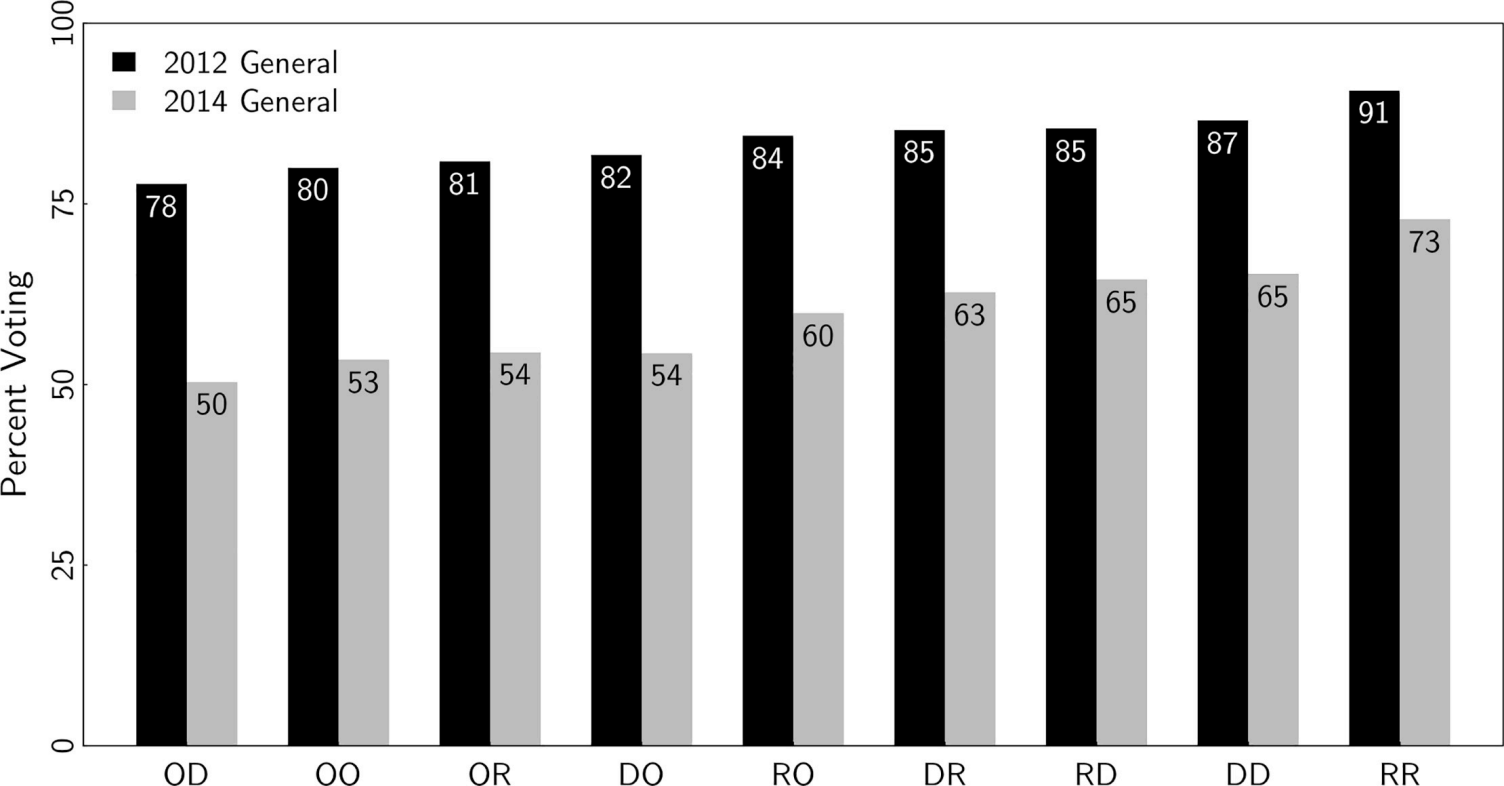
Individual turnout by household type, general elections 2012 and 2014. Note: The x-axis defines household type. The first letter represents the group being measured whereas the second letter represents the spouse. For instance, ‘OD’ represents independents married to Democrats, whereas ‘DO’ represents Democrats married to independents.

In Fig 3, we measure turnout conditional on household type. For Democrats, Republicans, and independents, we measure their turnout in the 2012 and 2014 general elections within each possible type of household. For the sake of simplicity of presentation, we leave out the primaries here, but the rank order of turnout by household type is the same for primaries as for general elections.

In the general elections, independents have the lowest turnout, no matter the party of their spouse. Turnout among Democrats and Republicans varies, in some cases substantially, by the party of their spouse. For Republicans married to independents, 60% voted in the 2014 general election. For Republicans married to Republicans, 73% voted.

Turnout appears to be more sensitive to the party of one’s spouse for Republicans compared to Democrats. A Democrat married to a Republican is two percentage points less likely to vote than a Democrat married to a fellow Democrat (2014 midterm). But a Republican married to a Democrat is eight percentage points less likely to vote than a Republican married to a Republican. It is difficult to know why the estimate for Republicans is four times larger. Turnout among R-R couples was unusually high in these elections compared to any other pairing. Perhaps, R-R households are more likely to be mobilized or more likely to possess other traits (e.g. older age, political interest) that makes them unusually likely to vote relative to other family units.

To see the relationship between partisanship, household type, and turnout more clearly, we model election turnout using hierarchical logistic regression. For each election type (2012 primary, 2012 general, 2014 primary, 2014 general), we model each individual *i*’s turnout as a function of his or her state of residence (indexed on *j*), race and gender combination (*k*), age (*l*), and household type (*m*):

$$\mathrm{Pr}\left(y_{i}=1\right)=logit^{-1}\;(\alpha^{0}+\alpha_{j[i]}^{state}+\alpha_{k[i]}^{race,gender}+\alpha_{l[i]}^{age}+\alpha_{m[i]}^{type})$$

The terms after the intercept (*α*^0^) are varying intercepts, where each batch of terms is drawn from a normal distribution with mean zero and an estimated variance, drawn from the data:

$$\alpha^{state}∼Normal(0,\sigma_{state}^{2})$$

$$\alpha^{race,gender}∼Normal(0,\sigma_{race,gender}^{2})$$

$$\alpha^{age}∼Normal(0,\sigma_{age}^{2})$$

$$\alpha^{type}∼Normal(0,\sigma_{type}^{2})$$

Each registered voter is in one of nine household types, (DD, DO, DR, OD, OO, OR, RD, RO, RR), where the first letter signifies the registrant’s party and the second letter signifies the party of the spouse. For each year of age, 18–90, and for each state, the relevant terms in the model act similarly to “fixed effects” in a standard logistic regression model. The model also includes the combination of gender-race pairs: white female, white male, black female, black male, Latina female, Latino male, other/unknown female, other/unknown male.

In S3 Table, we plot the logit regression coefficients for household type, race and gender, and state. In S3 Fig., we plot the coefficients on individual year of age. Here, we show tabular and graphical views of the key relationship between household type and turnout.

First, in Table 4, we show the differences in turnout among partisans depending on the partisanship of their spouses when, based on all other variables in the model, turnout is expected to be at 50%. For example, the upper-right number in the table, -0.12, has the following interpretation: For Republican registrants who, based on their state, age, gender, and race, were 50% likely to vote in a 2012 primary, they were 12 percentage points less likely to vote if their spouse was a Democrat than if their spouse was a Republican.

**Table 4:** Turnout differences by household type, open vs. closed primary state.

Turnout of this type	DO	DR	RO	RD
…compared to this type	DD		RR	
*2012 Primary*				
All	-0.13	-0.04	-0.17	-0.12
Open	-0.08	-0.03	-0.12	-0.10
Closed	-0.17	-0.05	-0.18	-0.12
*2012 General*				
All	-0.07	-0.03	-0.12	-0.10
Open	-0.06	-0.03	-0.11	-0.10
Closed	-0.07	-0.03	-0.12	-0.10
*2014 Primary*				
All	-0.14	-0.06	-0.15	-0.08
Open	-0.08	-0.04	-0.13	-0.08
Closed	-0.18	-0.07	-0.17	-0.09
*2014 General*				
All	-0.08	-0.03	-0.11	-0.09
Open	-0.06	-0.03	-0.10	-0.09
Closed	-0.08	-0.03	-0.11	-0.09

Note: Table show marginal turnout change at maximum part of logit curve. When turnout is expected to be 50% based on all other variables, the coefficients here represent the independent effect of household type. Open primary states in our sample are: AK, AZ, CA, ID, LA, MA, NC, NE, NH, OK, RI, SD, UT, WV in midterm years, and CA, ID, MA, NC, OK, RI, SD, UT, WV for presidential primaries. Closed primary states are: CO, CT, DE, DC, FL, IA, KS, KY, ME, MD, NV, NJ, NM, NY, OR, PA, WY in midterm years, and AK, AZ, CO, CT, DE, DC, FL, HI, IA, KS, KY, LA, ME, MD, NE, NV, NH, NJ, NM,NY, OR, PA, WA, WY for presidential primaries

When we re-run the turnout analysis for all thirty-two definitions of marriage, the estimates are always the same or larger in magnitude than in our main definition of marriage. For example, when we include same-sex couples and couples who have different surnames (but keep all the other marriage conditions the same), the Democrat married to a Republican is seven percentage points less likely to vote in the 2012 primary compared the Democrat married to a Democrat, three points larger than the -0.04 estimate in the table here.

The table shows the marginal effects for all states, and then separately for open and closed primary states. In both general elections, the results for open and closed primary states are similar. This is important because a different pool of registrants may choose to register as independents in an open or closed state (but see [23]). Open and closed systems do produce different results in primaries. In closed states, independents are ineligible to participate in primaries. In those states, partisans married to independents are considerably less likely to vote in primaries than partisans married to co-partisans.

**Fig 4:**
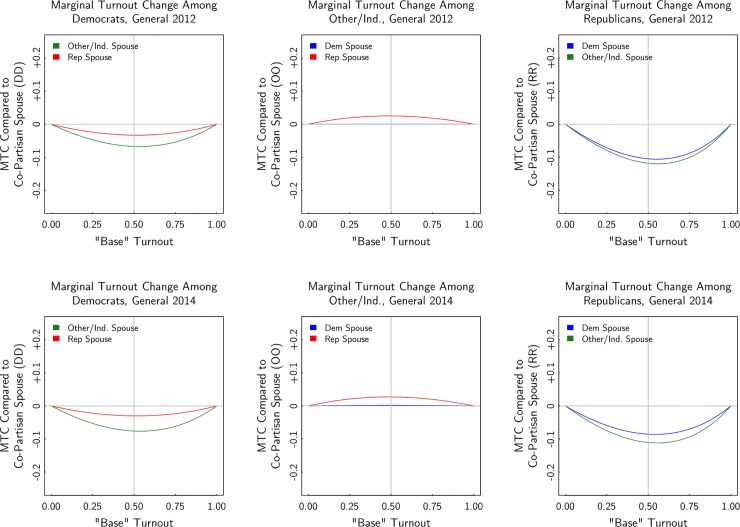
Estimated turnout of mixed-partisan marriage, 2012 and 2014 general elections.

**Fig 5:**
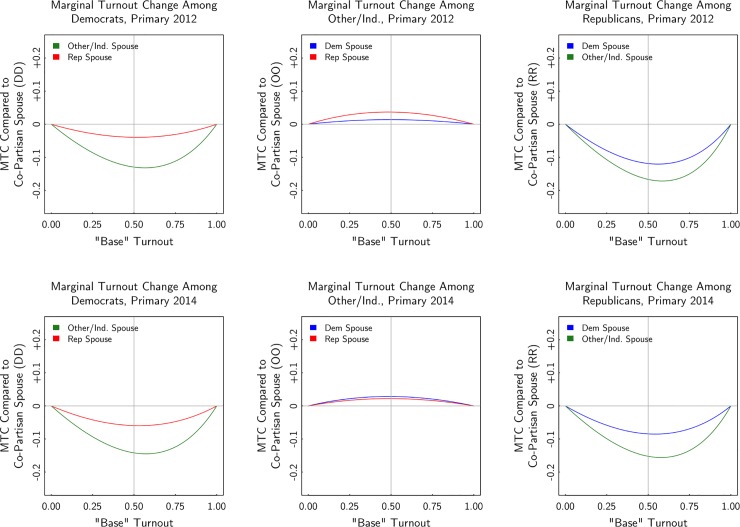
Estimated turnout of mixed-partisan marriage, 2012 and 2014 primary elections.

Figs 4 and 5 present the results graphically. Rather than just showing the estimate in the scenario where turnout is otherwise expected to be 50%, the figures show the estimate at each level of base turnout. Where the x-axis is 0.5, the estimated level is the same as shown in Table 4. In evaluating the results over the range of the x-axis, consider the turnout level of partisans and independents in each election, as reported in Table 3. In the presidential election, turnout among registrants (particularly among Democrats and Republicans) is well above 50%. In the midterm election, average turnout is close to 50%, and in the primaries, average turnout is lower.

There are two key takeaway points from the figures: (1) Controlling for age, state, race, and gender, the party affiliation of one’s spouse can bear a strong relationship to turnout; and (2) the relationship varies considerably by the party of the voter and the party of their spouse. This heterogeneity is surprising. Registered Democrats married to Democrats have turnout as much as six percentage points higher in the general elections and as much as 18 percentage points higher in the primary elections compared to Democrats married to independents. The estimated turnout difference between Democrats married to Democrats versus Democrats married to Republicans is considerably smaller than the difference between Democrats married to Democrats versus Democrats married to independents.

For registered Republicans, the results are different. First, the point estimates are about twice as large as they are for Democrats. For example, in the 2012 election, a Democrat married to an independent was up to 6 percentage points less likely to vote than a Democrat married to a Democrat, but a Republican married to an independent was up to 12 percentage points less likely to vote than a Republican married to another Republican. Second, unlike for Democrats, for Republicans the estimated differences in turnout for those married to independents versus to Democrats are similar. For Democrats, the turnout differences was larger in households in which the Democrats were married to independents.

For independent voters, the results are different still. Independents exhibit low levels of turnout and their turnout level doesn’t vary with the party affiliation of the spouse. As seen in the middle panels of Fig 4, independents married to partisans only have slightly higher turnout than independents married to independents. Note that the middle panels of Fig 5 should be interpreted with caution, as independent voters are not eligible to vote in primaries in some states. However, the estimates for independents are similar in primaries and in generals, as seen in the comparison between Figs 4 and 5.

Conventional wisdom in political science says that most independent voters are closet partisans. Many who identify and register as independent behave as partisans. (As Hersh notes [15], for example, when campaigns target registered independents, most of these targets are not individuals who would report on survey that they are “pure” independents or undecided.) In our analysis, however, we see that independents not only are different from partisans, but in households in which they are married to partisans, the partisans vote less than other partisans. The difference in partisan turnout rates in generals, and especially in primaries, depending on the affiliation of their spouse, is remarkable. Even after controlling for important correlates of turnout like race, state, and age, the partisanship of one’s spouse bears a strong relationship with voter turnout.

While a causal investigation is beyond the scope of this study, we note with interest the relationship in open versus closed primaries. In closed primaries, in marriages between a partisan and an independent, only the partisan is eligible to participate. It is in these elections in which the partisan married to an independent is least likely to cast a ballot (compared to partisans in other family units). The fact that these elections exhibit the strongest relationships with turnout lead us to wonder whether this is the result of co-habitation rather than (just) homophily. Sorting into marriages for reasons correlated with participation may explain the general election patterns, but perhaps the larger differences in closed primaries is the result of cohabitation. Of course, a cohabitation effect could take multiple forms. It could be that when one’s spouse is ineligible to vote, an eligible partisan is less likely to vote for reasons such as they don’t want to walk to the polling precinct alone. Or it could be something entirely different, like that campaigns mobilize households in which two members of a couple are eligible to vote rather than just one. Regardless, variations in participatory eligibility may offer an opportunity for future research to investigate the circumstances in which different family units participate or fail to participate in politics.

## Discussion

Students of political behavior have developed a rich set of findings about how individuals participate in politics. But individuals are commonly situated in households, in networks, in communities. Here, we take an important step forward in learning about the political behavior of the essential small group in a society: married couples. First, we have learned about the basic rate of party intermarriage in the United States. Seven in ten married couples share a party affiliation, three in ten do not. Of those who do not, two-thirds are partisans married to independents, and one third are partisans married to someone of the other political party.

Second, well-known relationships between partisanship and gender, partisanship and age, and partisanship and geography operate similarly within households as they operate between individuals. Women are not only more likely to be Democratic than men, but among married couples who differ in their partisanship, the female partner is twice as likely as the male partner to be the Democrat. Older voters are not only more likely than younger voters to identity as a partisan, but married couples age 80 are 66% more likely to be in a D-D or R-R marriage as compared to married couples age 30.

Across different neighborhoods, between 50% and 75% of married households are composed of two Democrats or two Republicans, and these households are least common in neighborhoods that split their votes evenly between Democratic and Republican candidates. This has an important implication for our understanding of partisan geography. When we envision a “battleground” neighborhood in which numbers of Democrats and Republicans are roughly equal, we might instinctively think of neighbors disagreeing with neighbors. Importantly, in these neighborhoods we witness the highest rate of married people disagreeing with their own partners. This is likely to lead to a more tempered political climate in America’s political battlegrounds than what one otherwise might expect.

Finally, we have shown that the party of one’s spouse bears a relationship to voter participation. Partisans who are married to out-partisans, and especially partisans who are married to independents, exhibit lower rates of turnout than partisans married to co-partisans. The difference in turnout is especially pronounced in primary elections. Evidence from prior research suggests this is partially the result of individuals who are less engaged in politics being more willing to marry out-partisans. It is likely partially the result of mixed-partisan relationships having the effect of depressing turnout. The cohabitation effect seems plausible as an explanation for driving down turnout among partisans in primary elections, though we caution against drawing any causal conclusions from our study. Regardless of the precise mechanism, after controlling for individual attributes like age, race, and gender, the party affiliation of one’s spouse bears a strong independent relationship on one’s propensity to vote.

One important way in which this study can be extended in the future is in combining survey research with government records. In recent years, common political science surveys like the NES and CCES have linked respondents to validated voter turnout data. On account of the importance of family networks to individual behaviors and attitudes, large surveys in the future should utilize voter registration records and commercial data to compose representative samples of households and to link representative samples of individuals to contextual information about their household. Another opportunity for future research is to connect over-time snapshots of voter registration databases into a panel. Questions about how party affiliation changes over time and correspond to differences in household structures may be answerable through such a design. The panel approach as well as integration between surveys and household contextual data would usefully build knowledge on this important but understudied area of scholarly research.

## Supporting information

S1 Table Percent in each household type, alternate definitions of marriage. (DOCX)

S2 Table Racial sorting and party sorting compared, southern states. CAPTION: Analysis restricted to three states with both racial registration and self-identified party affiliation (FL, LA, NC). The racial combinations include Whites (W), Blacks (B), Hispanics (H), and others (O). In all three versions of the marriage definition shown here, married couples are restricted to pairs who are within fifteen years of age. In households with 2–10 registered voters, the oldest eligible pair are observed.
(DOCX)

S3 Table Regression table. (DOCX)

S1 Text Evaluating the rate of interpartisan marriage. (DOCX)

S1 Fig Random pairing vs. perfect sorting vs. reality. CAPTION: Black lines indicate percent of married couples who are both Democrats or both Republicans among their age and geographic cohort. The upper bound represents the share that would be same-party if partisans always married each other. The lower bound represents the share of same-party if marriage was random with respect to partisanship.
(TIFF)

S2 Fig Age distribution of married couples. (TIFF)

S3 Fig Regression results: Varying intercepts on individual years of age. (TIFF)
