# Supplementary material for: Mixed partisan households and electoral participation in the United States
Source: PLoS One. 2018 Oct 10;13(10):e0203997. doi: 10.1371/journal.pone.0203997 (PMC6179382; doi:10.1371/journal.pone.0203997)
Supplement: S1 Table — (DOCX) [file pone.0203997.s001.docx]

**S1 Table: Percent in each household type, alternate definitions of marriage**

| **Restrictions** | | | | |  | **Percent in Combinations** | | | | | |
| --- | --- | --- | --- | --- | --- | --- | --- | --- | --- | --- | --- |
| Same | HH Size | Male/ | Age w/in | Oldest or | Num. |  | | | | | |
| Surname | ≤ 10 | Female | 15 yrs. | Random | Households | DD | OO | RR | DO | RO | DR |
| Yes | Yes | Yes | Yes | Oldest | 18,275,001 | 25 | 15 | 31 | 10 | 9 | 10 |
| No | Yes | Yes | Yes | Oldest | 23,387,150 | 26 | 15 | 26 | 13 | 10 | 11 |
| Yes | No | Yes | Yes | Oldest | 18,276,005 | 25 | 15 | 31 | 10 | 9 | 10 |
| No | No | Yes | Yes | Oldest | 23,415,393 | 26 | 15 | 26 | 13 | 10 | 11 |
| Yes | Yes | No | Yes | Oldest | 19,611,954 | 25 | 15 | 30 | 11 | 9 | 10 |
| No | Yes | No | Yes | Oldest | 26,480,899 | 27 | 15 | 24 | 14 | 10 | 11 |
| Yes | No | No | Yes | Oldest | 19,612,989 | 25 | 15 | 30 | 11 | 9 | 10 |
| No | No | No | Yes | Oldest | 26,511,503 | 27 | 15 | 24 | 14 | 10 | 11 |
| Yes | Yes | Yes | No | Oldest | 20,410,653 | 25 | 14 | 29 | 11 | 9 | 11 |
| No | Yes | Yes | No | Oldest | 26,412,178 | 26 | 14 | 24 | 14 | 10 | 11 |
| Yes | No | Yes | No | Oldest | 20,411,658 | 25 | 14 | 29 | 11 | 9 | 11 |
| No | No | Yes | No | Oldest | 26,440,472 | 26 | 14 | 24 | 14 | 10 | 11 |
| Yes | Yes | No | No | Oldest | 23,186,027 | 26 | 15 | 27 | 12 | 9 | 11 |
| No | Yes | No | No | Oldest | 31,326,757 | 27 | 14 | 22 | 15 | 11 | 11 |
| Yes | No | No | No | Oldest | 23,187,062 | 26 | 15 | 27 | 12 | 9 | 11 |
| No | No | No | No | Oldest | 31,357,361 | 27 | 14 | 22 | 15 | 11 | 11 |
| Yes | Yes | Yes | Yes | Random | 18,275,001 | 25 | 15 | 30 | 10 | 9 | 10 |
| No | Yes | Yes | Yes | Random | 23,387,150 | 26 | 15 | 25 | 13 | 10 | 11 |
| Yes | No | Yes | Yes | Random | 18,276,005 | 25 | 15 | 30 | 10 | 9 | 10 |
| No | No | Yes | Yes | Random | 23,415,393 | 26 | 15 | 25 | 13 | 10 | 11 |
| Yes | Yes | No | Yes | Random | 19,611,954 | 25 | 15 | 29 | 11 | 9 | 11 |
| No | Yes | No | Yes | Random | 26,480,899 | 26 | 15 | 23 | 15 | 10 | 11 |
| Yes | No | No | Yes | Random | 19,612,989 | 25 | 15 | 29 | 11 | 9 | 11 |
| No | No | No | Yes | Random | 26,511,503 | 26 | 15 | 23 | 15 | 10 | 11 |
| Yes | Yes | Yes | No | Random | 20,410,653 | 25 | 15 | 28 | 12 | 9 | 11 |
| No | Yes | Yes | No | Random | 26,412,178 | 25 | 15 | 23 | 15 | 10 | 12 |
| Yes | No | Yes | No | Random | 20,411,658 | 25 | 15 | 28 | 12 | 9 | 11 |
| No | No | Yes | No | Random | 26,440,472 | 25 | 15 | 23 | 15 | 10 | 12 |
| Yes | Yes | No | No | Random | 23,186,027 | 25 | 15 | 26 | 13 | 9 | 12 |
| No | Yes | No | No | Random | 31,326,757 | 26 | 15 | 21 | 16 | 10 | 12 |
| Yes | No | No | No | Random | 23,187,062 | 25 | 15 | 26 | 13 | 9 | 12 |
| No | No | No | No | Random | 31,357,361 | 26 | 15 | 21 | 16 | 10 | 12 |
