# Supplementary material for: Mixed partisan households and electoral participation in the United States
Source: PLoS One. 2018 Oct 10;13(10):e0203997. doi: 10.1371/journal.pone.0203997 (PMC6179382; doi:10.1371/journal.pone.0203997)
Supplement: S2 Table — CAPTION: Analysis restricted to three states with both racial registration and self-identified party affiliation (FL, LA, NC). The racial combinations include Whites (W), Blacks (B), Hispanics (H), and others (O). In all three versions of the marriage definition shown here, married couples are restricted to pairs who are within fifteen years of age. In households with 2–10 registered voters, the oldest eligible pair are observed. (DOCX) [file pone.0203997.s002.docx]

**Table S2: Racial sorting and party sorting compared, southern states**

| Same | Male/ | **Percent in Party Combinations** | | | | | |  |
| --- | --- | --- | --- | --- | --- | --- | --- | --- |
| Surname? | Female | DD | OO | RR | DO | RO | DR |  |
| Yes | Yes | 25 | 12 | 34 | 10 | 9 | 11 |  |
| No | Yes | 25 | 13 | 28 | 12 | 10 | 12 | |
| Yes | No | 25 | 13 | 32 | 10 | 9 | 11 | |
| No | No | 26 | 13 | 26 | 14 | 10 | 12 |  |

| Same | Male/ | **Percent in Racial Combinations** | | | | | | | | | | |  | |
| --- | --- | --- | --- | --- | --- | --- | --- | --- | --- | --- | --- | --- | --- | --- |
| Surname? | Female | WW | BB | HH | OO | WB | WH | WO | BH | BO | HO |  | |  |
| Yes | Yes | 78 | 11 | 4 | 1 | 1 | 3 | 1 | 0 | 0 | 0 |  | |  |
| No | Yes | 72 | 12 | 6 | 1 | 3 | 4 | 2 | 1 | 0 | 0 |  | |  |
| Yes | No | 76 | 12 | 5 | 1 | 1 | 3 | 1 | 0 | 0 | 0 |  | |  |
| No | No | 69 | 14 | 6 | 1 | 3 | 4 | 2 | 1 | 0 | 0 |  | |  |
