# Supplementary material for: Mixed partisan households and electoral participation in the United States
Source: PLoS One. 2018 Oct 10;13(10):e0203997. doi: 10.1371/journal.pone.0203997 (PMC6179382; doi:10.1371/journal.pone.0203997)
Supplement: S3 Table — (DOCX) [file pone.0203997.s003.docx]

**Table S3: Regression table**

|  | Turnout 2012 | | Turnout 2014 | |
| --- | --- | --- | --- | --- |
| Ind Vars. | Primary | General | Primary | General |
| (Intercept) | -1.77 | 0.97 | -2.23 | -0.25 |
| DD | 0.30 | 0.20 | 0.53 | 0.20 |
| DO | -0.23 | -0.07 | -0.05 | -0.11 |
| DR | 0.14 | 0.07 | 0.29 | 0.08 |
| OD | -0.65 | -0.32 | -0.58 | -0.31 |
| OO | -0.71 | -0.32 | -0.70 | -0.31 |
| OR | -0.56 | -0.22 | -0.61 | -0.20 |
| RD | 0.49 | 0.10 | 0.36 | 0.14 |
| RO | 0.28 | 0.04 | 0.07 | 0.04 |
| RR | 0.97 | 0.52 | 0.70 | 0.49 |
| White Female | 0.29 | 0.35 | 0.25 | 0.39 |
| White Male | 0.29 | 0.24 | 0.27 | 0.44 |
| Black Female | 0.38 | 0.49 | 0.41 | 0.35 |
| Black Male | 0.11 | 0.06 | 0.20 | 0.08 |
| Latino Female | -0.27 | -0.08 | -0.36 | -0.30 |
| Latino Male | -0.31 | -0.34 | -0.40 | -0.33 |
| Oth/Unk. Female | -0.22 | -0.32 | -0.17 | -0.32 |
| Oth/Unk. Male | -0.26 | -0.39 | -0.20 | -0.30 |
| AK | 0.90 | -0.18 | 2.38 | 0.66 |
| AZ | 0.48 | -0.13 | 0.95 | -0.23 |
| CA | 0.81 | 0.17 | 1.01 | -0.15 |
| CO | -0.29 | 0.55 | 0.55 | 0.79 |
| CT | -0.59 | 0.00 | -1.15 | 0.21 |
| DC | -0.08 | -0.85 | 0.65 | -0.67 |
| DE | -0.46 | -0.22 | -0.74 | -0.59 |
| FL | 0.38 | -0.20 | 0.13 | -0.07 |
| IA | -1.07 | 0.28 | -0.28 | 0.31 |
| ID | 0.30 | 1.80 | 0.97 | 0.24 |
| KS | 0.07 | -0.25 | 0.49 | 0.11 |
| KY | -0.64 | -0.88 | 0.80 | -0.23 |
| LA | -0.59 | -0.08 | -11.86 | 0.56 |
| MA | 0.43 | 0.43 | 0.57 | 0.35 |
| MD | -0.32 | -0.06 | 0.39 | -0.10 |
| ME | -0.46 | -0.02 | -0.06 | 0.68 |
| NC | 1.17 | 0.13 | 0.30 | -0.03 |
| NE | 0.22 | -0.08 | 1.00 | -0.11 |
| NH | 1.38 | 0.20 | 0.48 | 0.18 |
| NJ | -0.89 | -0.37 | -0.78 | -0.61 |
| NM | -0.05 | -0.19 | 0.15 | -0.15 |
| NV | -0.45 | 0.31 | 0.25 | -0.38 |
| NY | -1.98 | -0.62 | -1.05 | -0.66 |
| OK | 0.34 | -0.33 | 0.52 | -0.54 |
| OR | 0.77 | 0.43 | 1.16 | 0.63 |
| PA | -0.20 | -0.15 | 0.00 | -0.38 |
| RI | -0.06 | -0.21 | 1.14 | 0.17 |
| SD | -0.55 | -0.25 | 0.26 | -0.11 |
| UT | 0.06 | 0.34 | -0.18 | -0.41 |
| WV | 0.61 | -0.72 | 0.50 | -0.47 |
| WY | 0.78 | 1.13 | 1.69 | 1.00 |
