# Supplementary material for: Mixed partisan households and electoral participation in the United States
Source: PLoS One. 2018 Oct 10;13(10):e0203997. doi: 10.1371/journal.pone.0203997 (PMC6179382; doi:10.1371/journal.pone.0203997)
Supplement: S1 Text — (DOCX) [file pone.0203997.s004.docx]

# S1 Text: Evaluating the rate of partisan intermarriage

An auxiliary question in this analysis is whether the observed rate of partisan intermarriage is higher or lower than what might be expected. What is the appropriate baseline expectation? For one, we can compare to marital sorting on race. In a 2012 analysis, the Pew Research Center estimated that 8% of married couples were racially or ethnically intermarried.^[[1]](#footnote-1)^ If 92% of couples marry within their racial/ethnic group, conventional wisdom would suggest a considerably lower rate of same-party marriage than same-race marriage. For many Americans, party affiliation is likely to be a less salient characteristic in mate selection than race. On the other hand, in recent research on partisan affect, Iyengar and Westwood (2015) argue that “the level of partisan animus in the American public exceeds racial hostility,” which suggests that perhaps partisan sorting might be on par with racial sorting in contemporary U.S. marriages.

Like race, party is wrapped up with other characteristics that individuals use in their selection of mates. If one’s mating pool is restricted to individuals who are roughly the same age, in the same geographic vicinity, in the same socio-economic stratum, perhaps in the same religion, there might be a low rate of inter-party marriage simply because the available pool of mates is incidentally politically homogenous. Thus, in addition to comparing inter-party marriages with inter-racial marriages, we also evaluate the rate of inter-party marriages by looking within an individual’s pool of potential mates who share an age and a geographic area. We compare the observed rate of inter-party marriage to the rate of inter-party marriage that would exist if individuals chose randomly from their age-geography pool of potential partners.

Consider the following exercise. Suppose that we define for each person a pool of potential partners. These partners are the same age and live in the same city. We first ask: what percentage of these potential partners are Democrats or Republicans? For instance, suppose that among married, registered individuals who are 32 years old living in Chicago, 70% are Democrats. If Democrats only married other Democrats, then 70% of the married couples would also be Democrats. If Democrats married randomly across partisan groups within their age-geography cohort, then in expectation 49% of married couples (*.*7 × *.*7) would be Democratic-only couples.

In Fig. S1, we generate bounds for each age group. The upper bound represents the share of married couples that would be Democratic-only or Republican-only if all registrants only married people who shared their party affiliation. The lower bound represents the share that would be Democratic-only or Republican-only if marriages were random with respect to partisanship. In the first row of plots, we show the national average (and do not account for the geographic location of potential pairs). Then, as illustrations, we show two cities, New York and Miami, where we restrict the pool of potential pairs to individuals who are the same age and also living in that city.

Consider the results in Fig. S1 in comparison to Table 1. For example, according to the table, 30% of married couples are Republican-only. Observe the lower bound in the upper-right plot in Fig. S1. If partners were choosing each other from the pool of people their age, and if they did not consider party at all, we would see about 20% of married couples in the country being Republican only, just by chance. On the other hand, looking at the upper-bound, if everyone married someone who shared their party affiliation, about 40% of couples would be Republican-only.

The results suggest both that there are more same-party marriages than if couples did not sort by party at all and also that there are many fewer same-party marriages than if partisans married only other partisans. Obviously, individuals are selecting mates based on more factors than age and location, and many of those factors are likely to be correlated with partisanship. Thus, in claiming that there is more sorting into same-party relationships than expected by chance, we do not mean to imply that couples are explicitly selecting on partisanship. But the combination of partisanship and attributes correlated with partisanship clearly generate more same-party marriages than if people were merely marrying randomly within their age-geography pool.

Perhaps a more intuitive way to anchor one’s interpretation of the rate of partisan intermarriage is to compare this with racial intermarriage. To do so, we focus on the three southern states where voters are registered by party and where voters are also listed in the public record with their racial identity (See Hersh (2015), Ch 6.). In Table S2, we observe racial and partisan intermarriage in these states, showing results for three variants on our definition of marriage. In the second row, we allow for couples who have different surnames. In the third row, we also allow same-sex couples.

With marriage defined as in the first row, 71% of couples are in same-party marriages. In comparison, 93% of compared are in same-race marriages. This registration-based estimate of same-race marriages is quite close to the 92% cited above from a 2012 Pew study. Compared to the 11% who are in Democratic-Republican households, only 4% are in White-Black, White-Hispanic, or Black-Hispanic households. While there are many more inter-party couples than inter-race couples, part of the reason why these numbers are different is because the racial composition is more predominantly of one category (white) than the party com- position is of any one category. Nevertheless, in the southern states that collect race and party, mixed-party couples are three times more common than mixed-race couples.

1. Pew research Center, “The Rise of Intermarriage,” February 16, 2012. http://www.pewsocialtrends.org/2012/02/16/the-rise-of-intermarriage/. [↑](#footnote-ref-1)
