# Supplementary figures and images for: Mixed partisan households and electoral participation in the United States
Source: PLoS One. 2018 Oct 10;13(10):e0203997. doi: 10.1371/journal.pone.0203997 (PMC6179382; doi:10.1371/journal.pone.0203997)

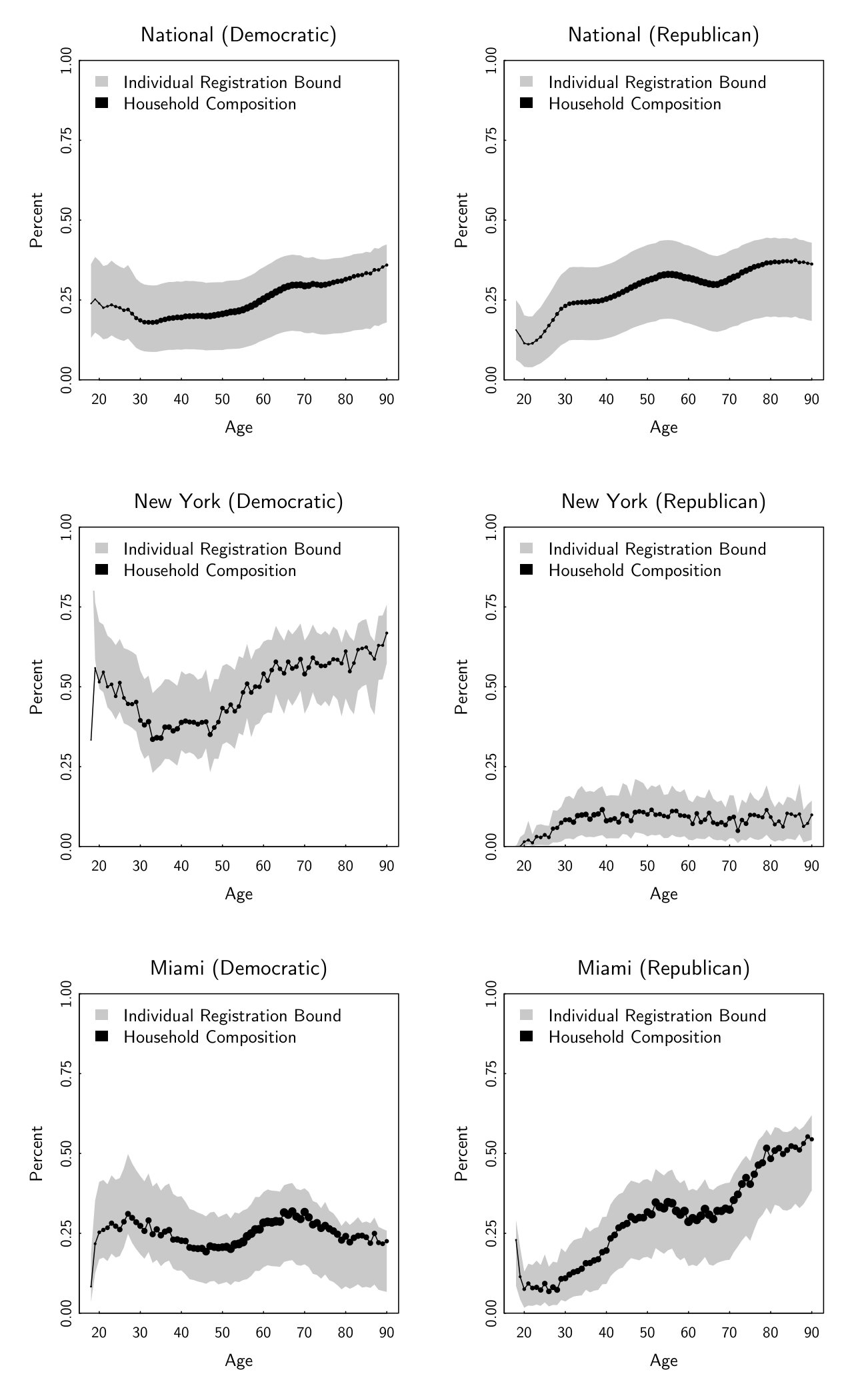

Supplement: S1 Fig — CAPTION: Black lines indicate percent of married couples who are both Democrats or both Republicans among their age and geographic cohort. The upper bound represents the share that would be same-party if partisans always married each other. The lower bound represents the share of same-party if marriage was random with respect to partisanship. (TIFF) [file pone.0203997.s005.tiff]

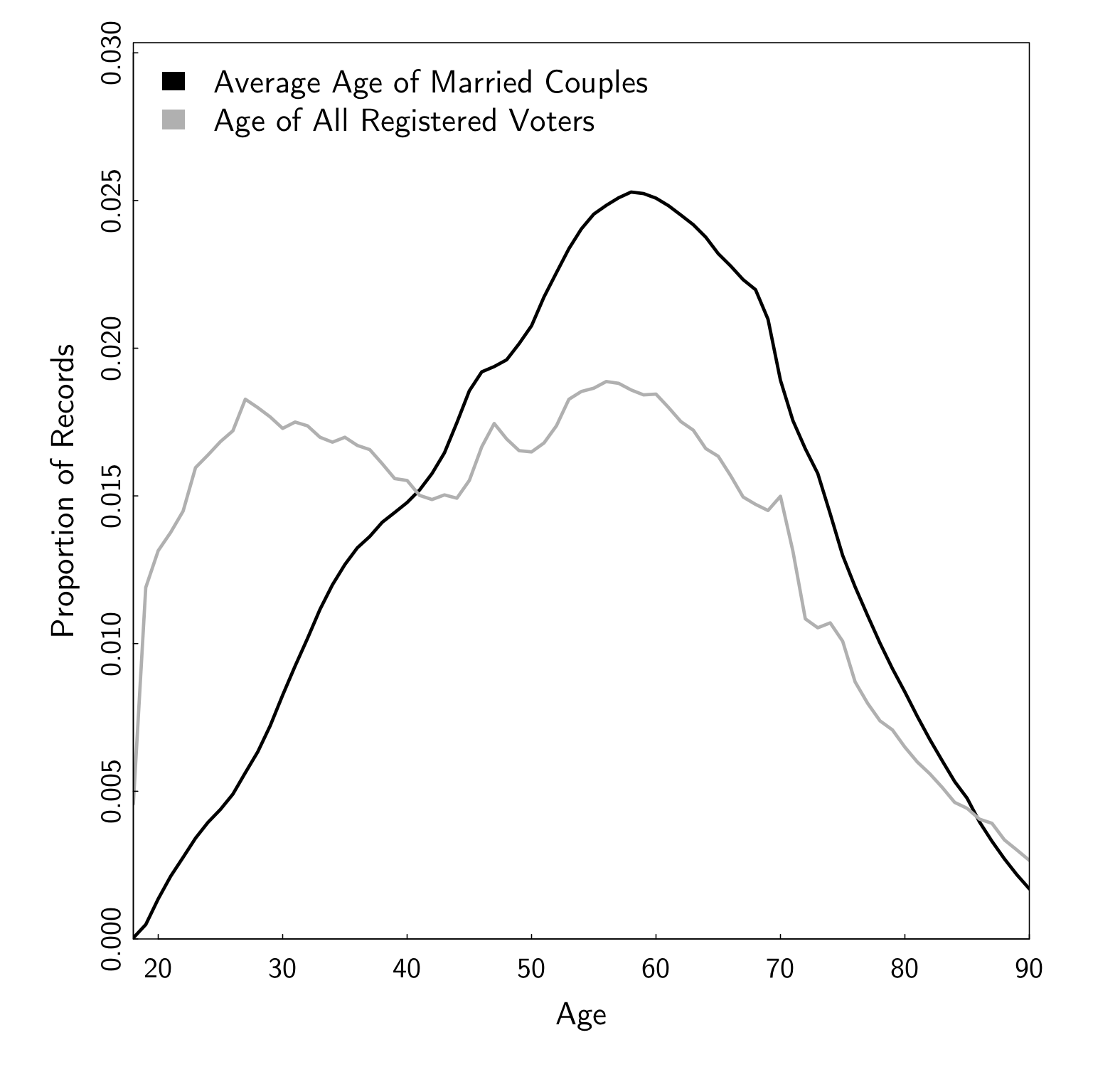

Supplement: S2 Fig — (TIFF) [file pone.0203997.s006.tiff]

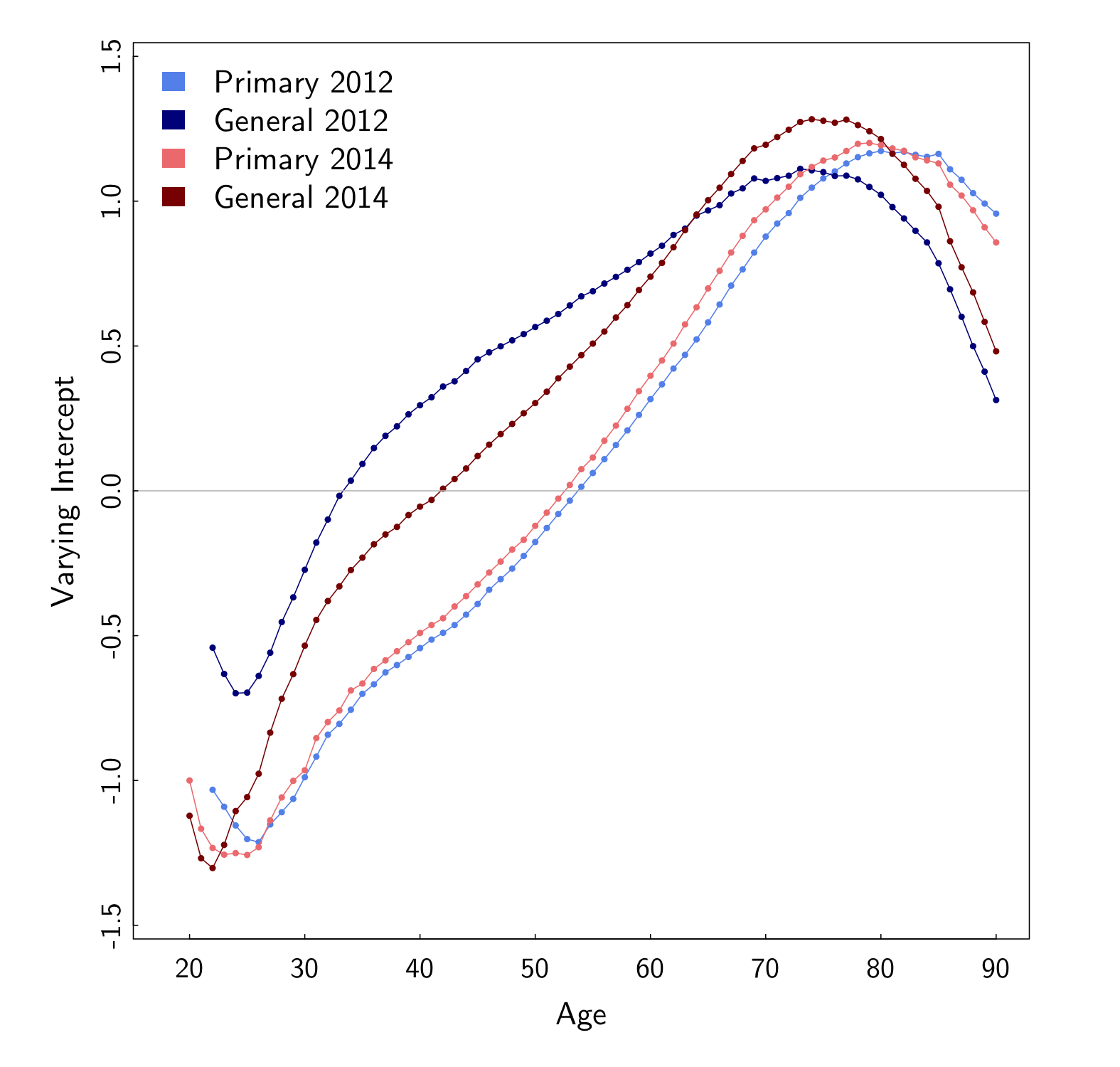

Supplement: S3 Fig — (TIFF) [file pone.0203997.s007.tiff]
